# Supplementary figures and images for: SAFB regulates hippocampal stem cell fate by targeting Drosha to destabilize Nfib mRNA
Source: eLife. 2024 May 9;13:e74940. doi: 10.7554/eLife.74940 (PMC11149935; doi:10.7554/eLife.74940)

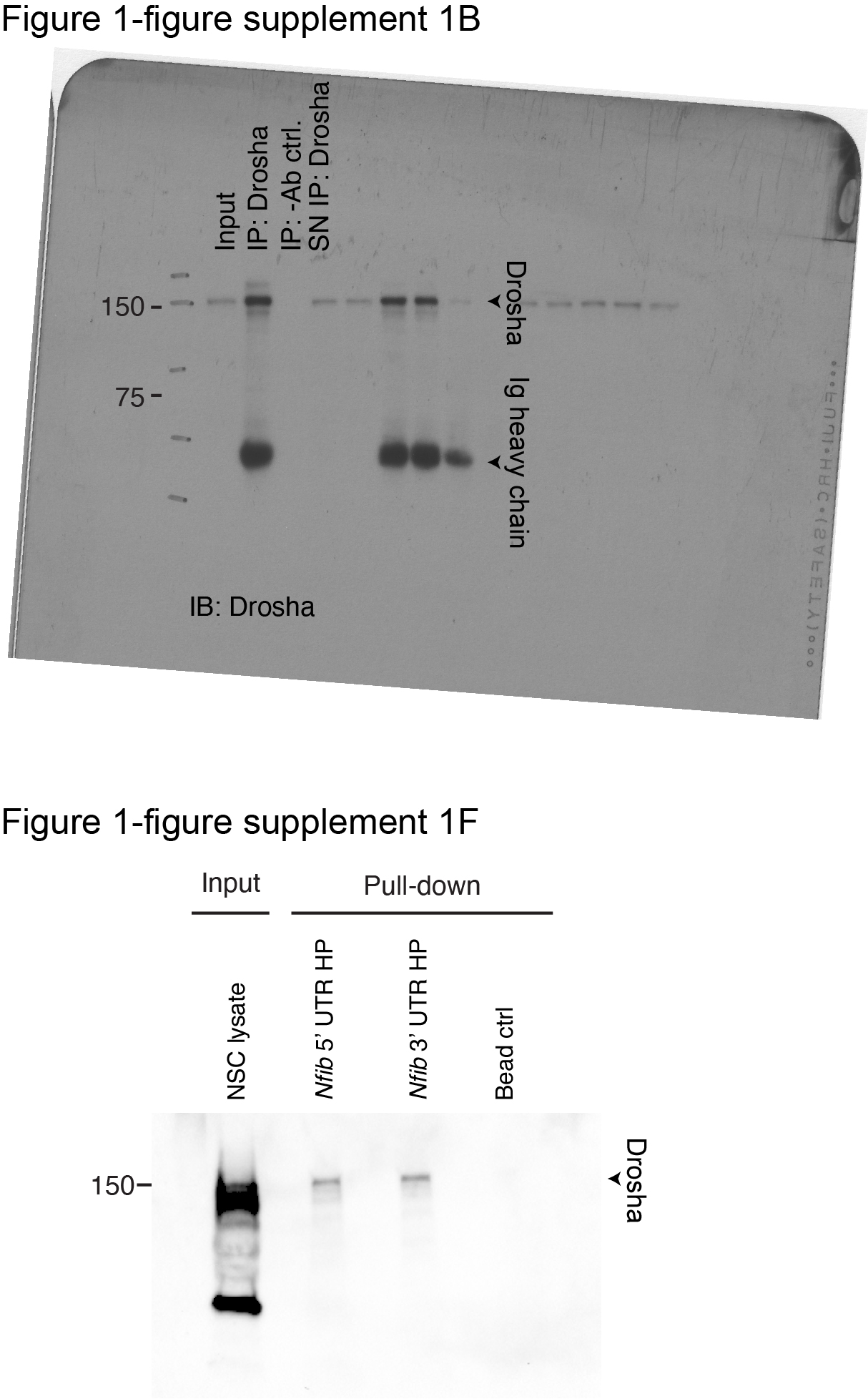

Supplement: Figure 1—figure supplement 1—source data 1. [file elife-74940-fig1-figsupp1-data1.zip › Figure 1-figure supplement 1 - source data.jpg]

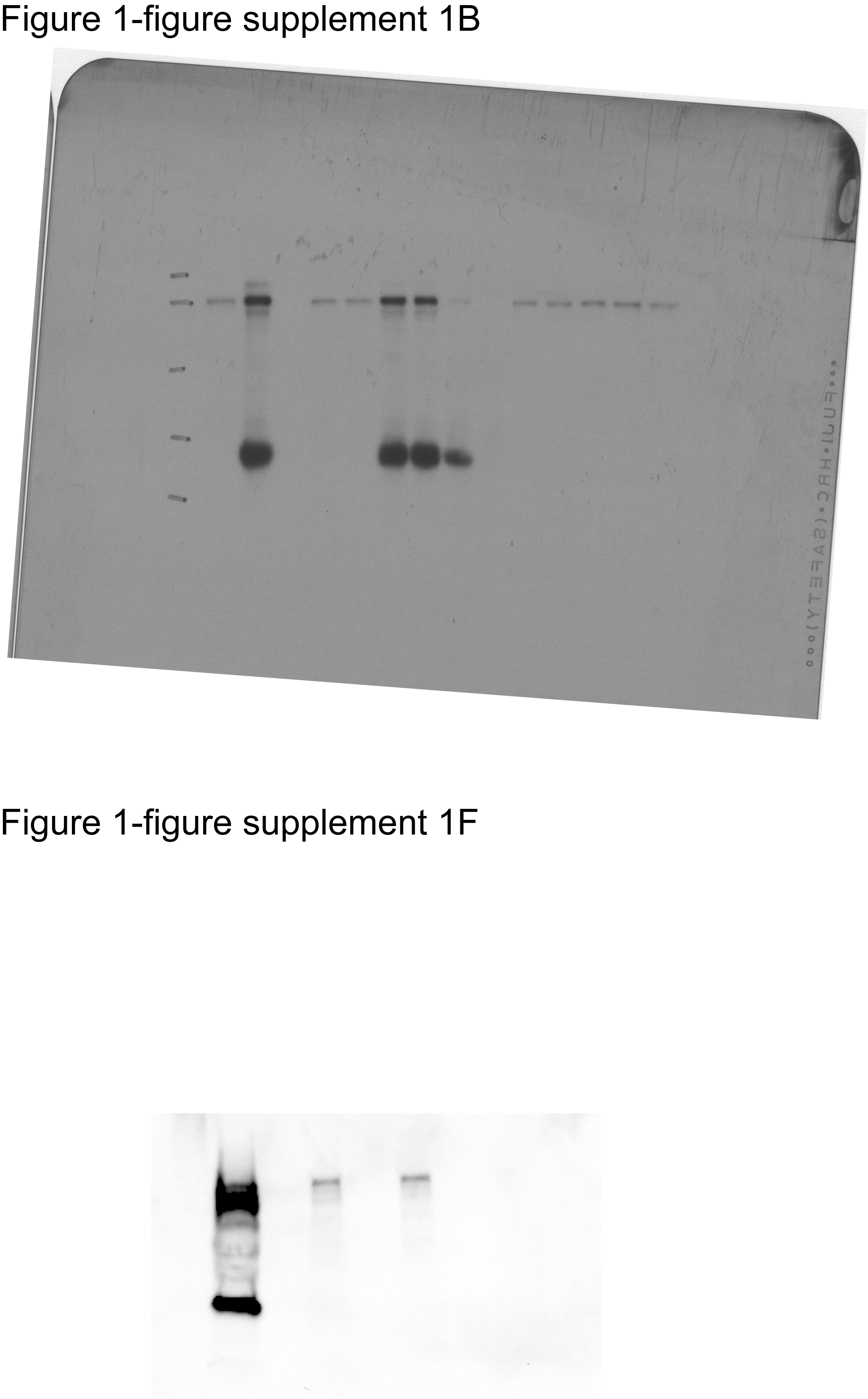

Supplement: Figure 1—figure supplement 1—source data 2. — Unlabelled. [file elife-74940-fig1-figsupp1-data2.zip › Figure 1-figure supplement 1 - source data.tif]

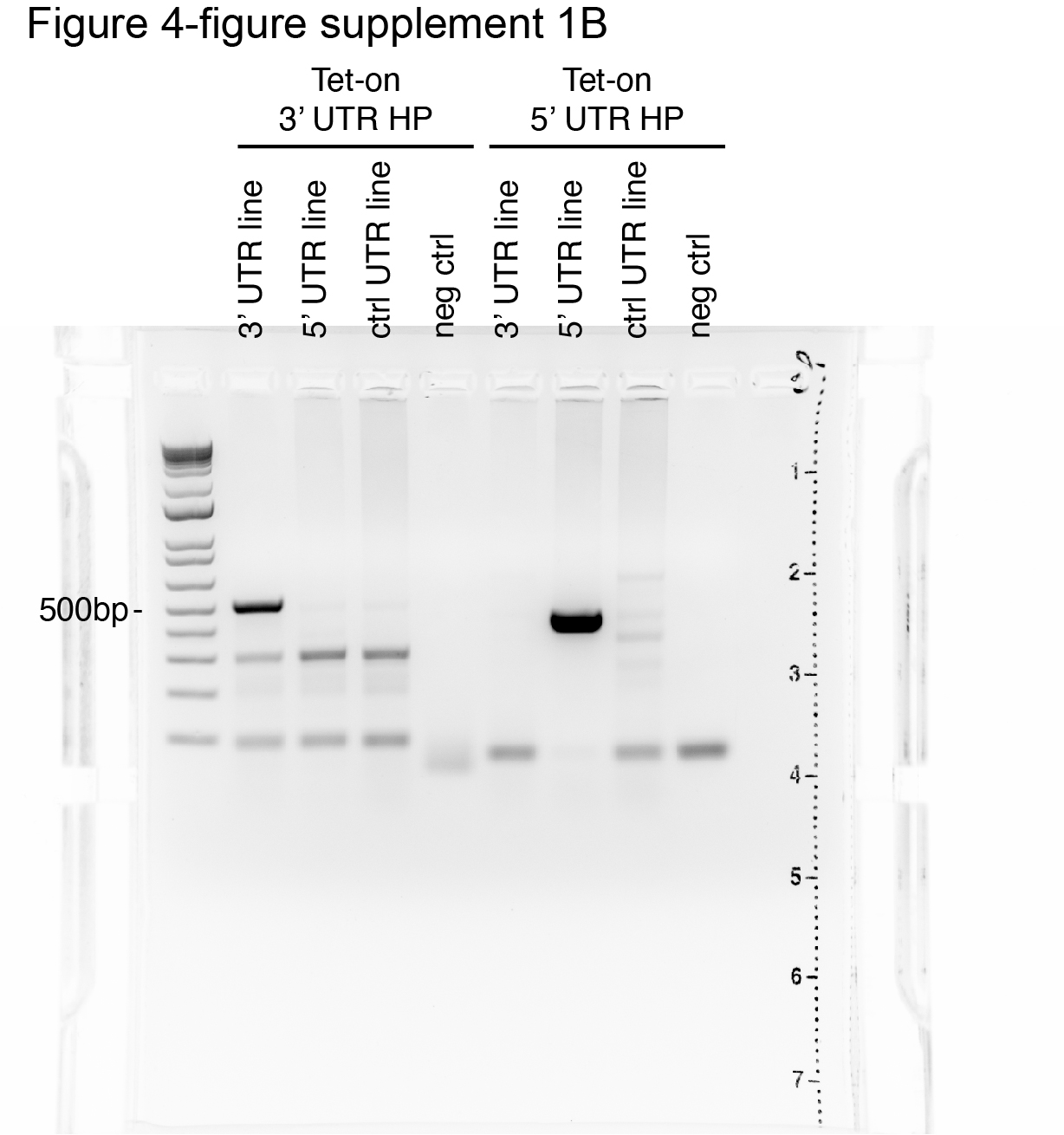

Supplement: Figure 4—figure supplement 1—source data 1. [file elife-74940-fig4-figsupp1-data1.zip › Figure 4-figure supplement 1 - source data.jpg]

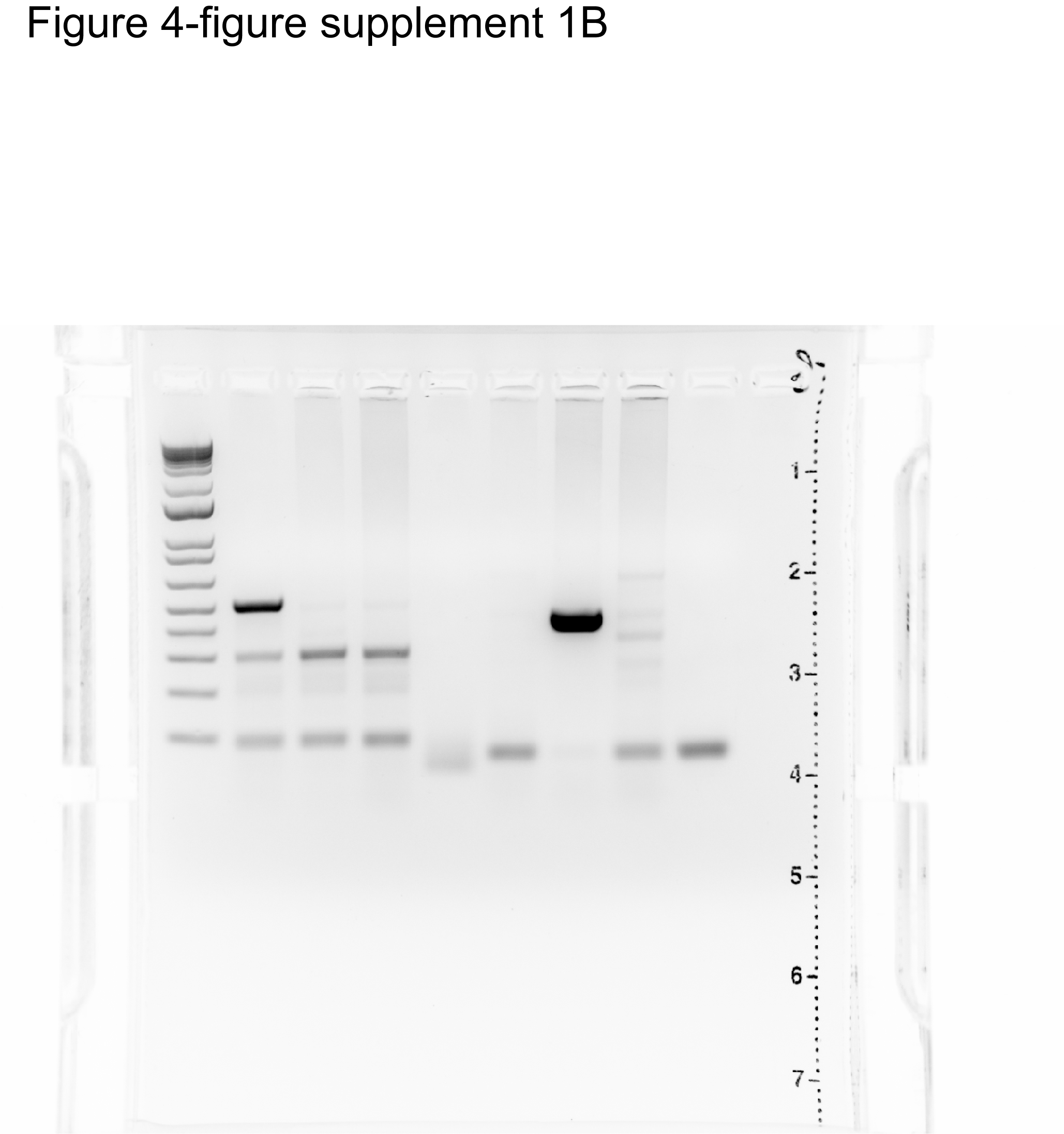

Supplement: Figure 4—figure supplement 1—source data 2. — Unlabelled. [file elife-74940-fig4-figsupp1-data2.zip › Figure 4-figure supplement 1 - source data.tif]

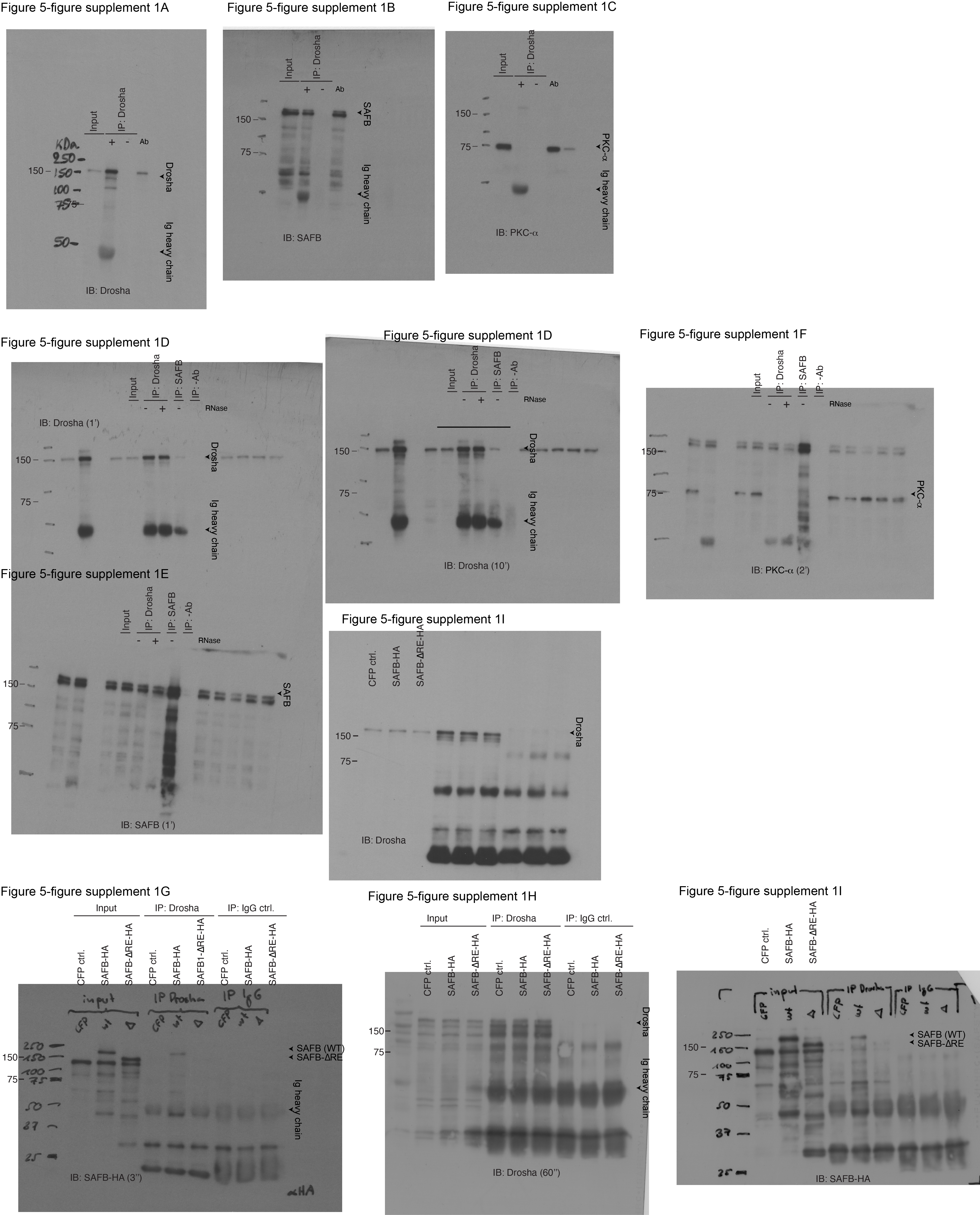

Supplement: Figure 5—source data 1. [file elife-74940-fig5-data1.zip › Figure 5 and Figure 5-figure supplement 1 - source data.jpg]

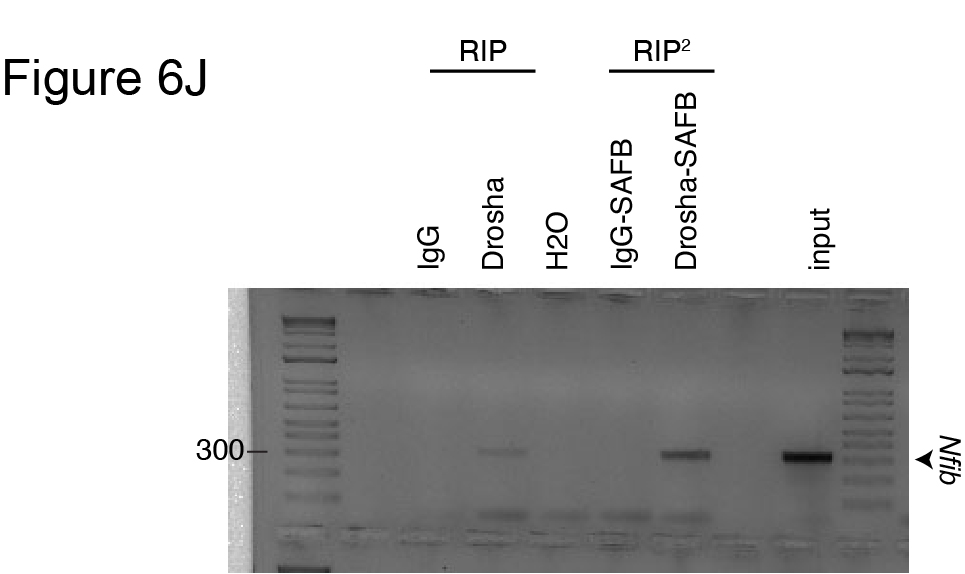

Supplement: Figure 6—source data 1. [file elife-74940-fig6-data1.zip › Figure 6 - source data.jpg]

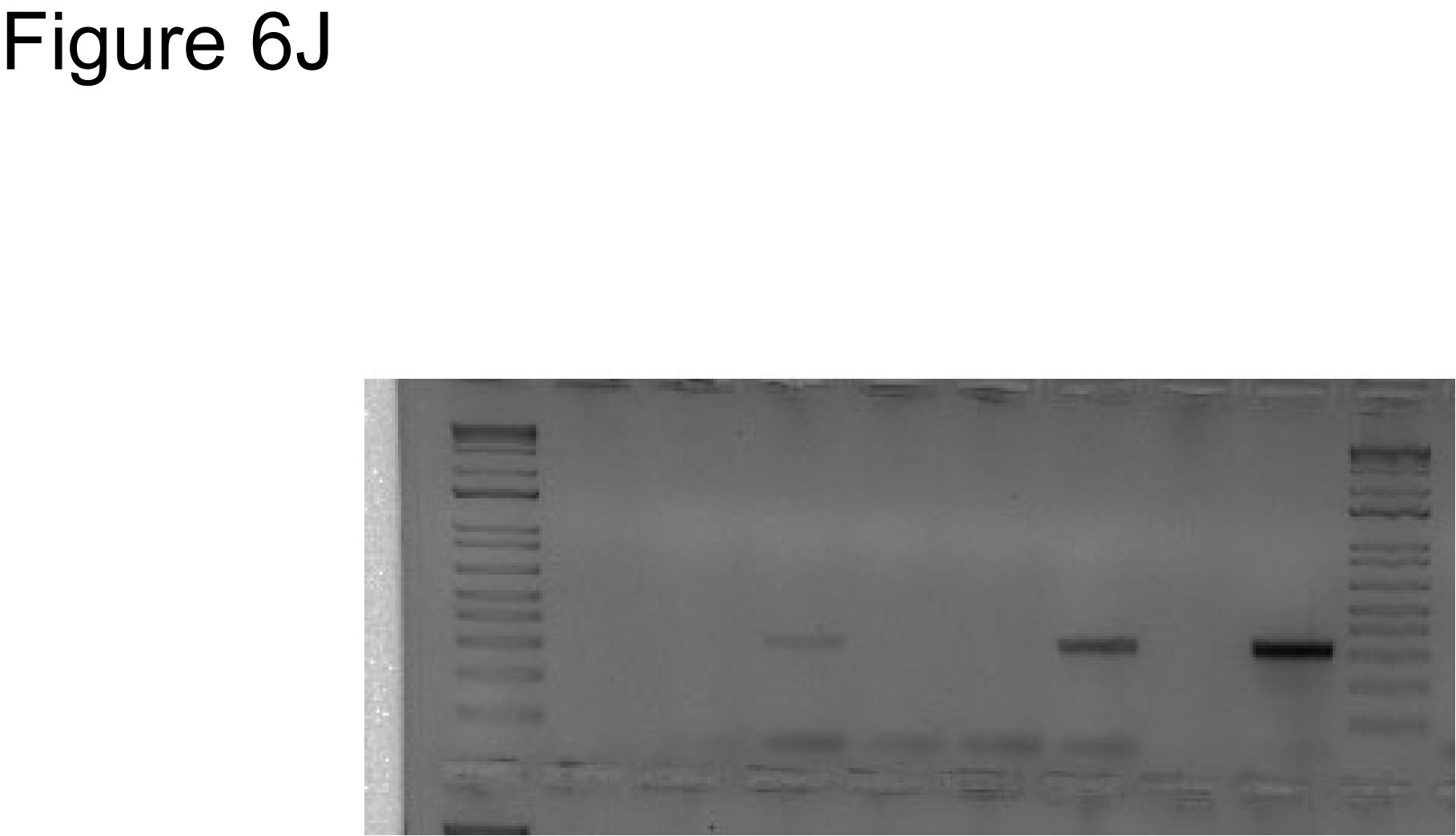

Supplement: Figure 6—source data 2. — Unlabelled. [file elife-74940-fig6-data2.zip › Figure 6 - source data.tif]

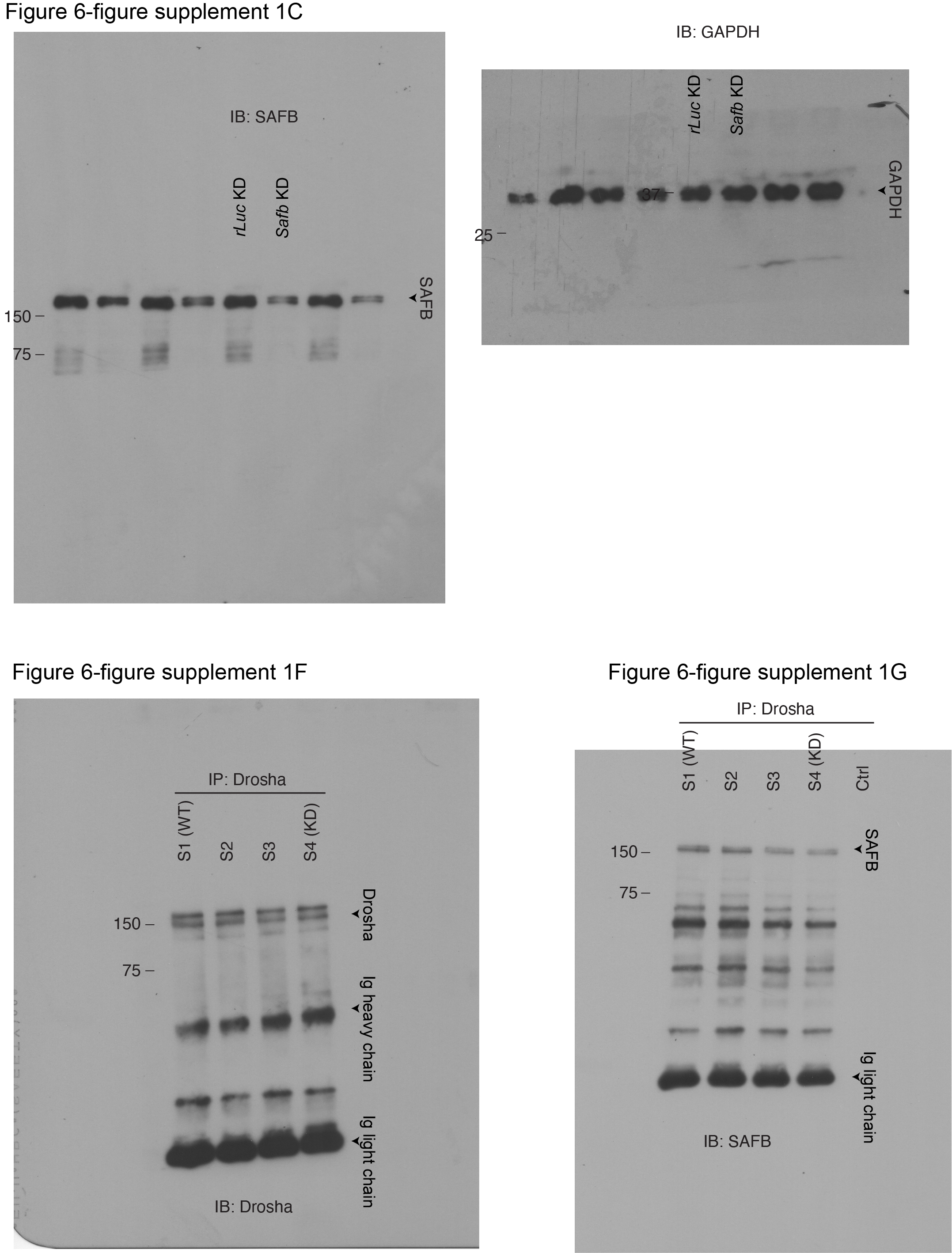

Supplement: Figure 6—figure supplement 1—source data 1. [file elife-74940-fig6-figsupp1-data1.zip › Figure 6-figure supplement 1 - source data.jpg]

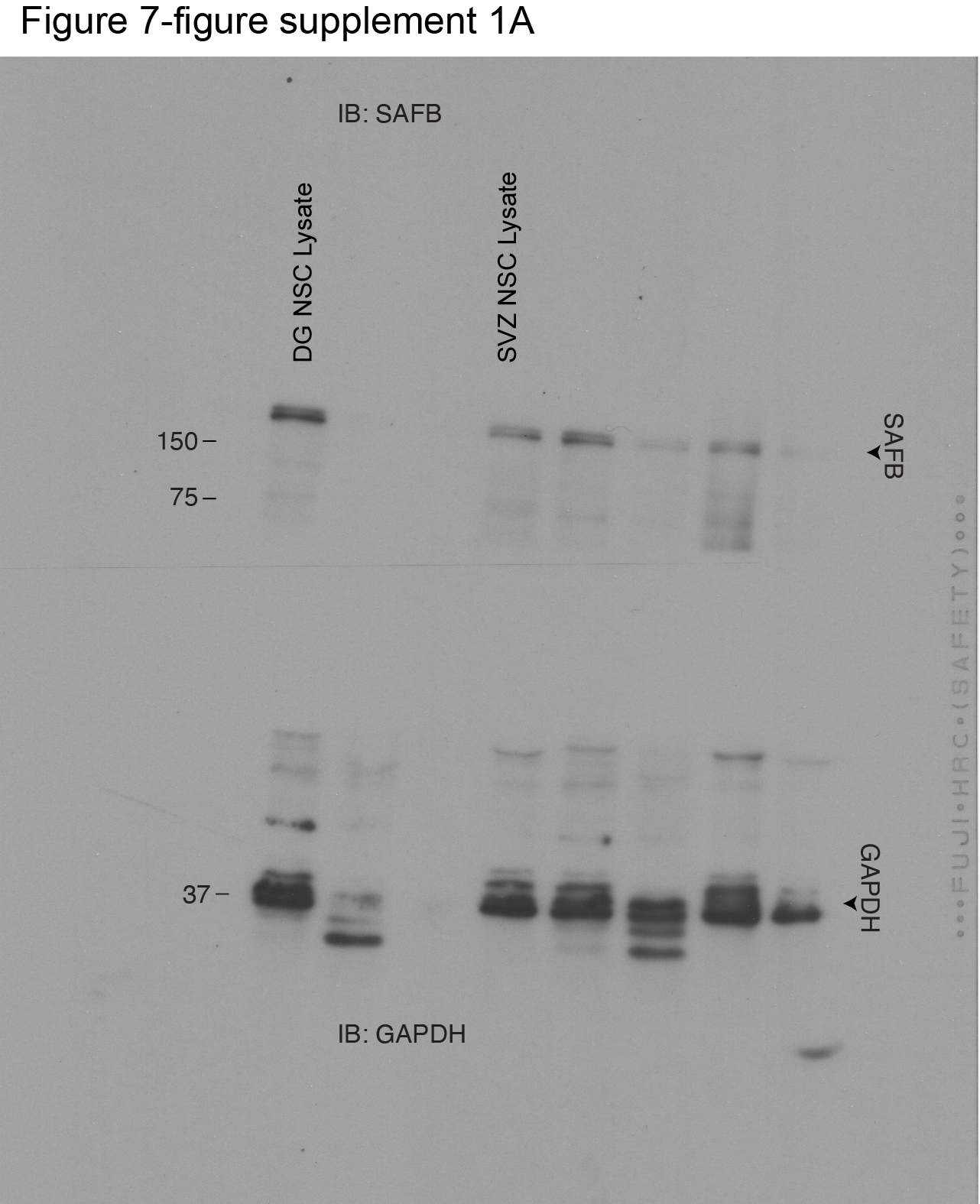

Supplement: Figure 7—figure supplement 1—source data 1. [file elife-74940-fig7-figsupp1-data1.zip › Figure 7-figure supplement 1 - source data.jpg]

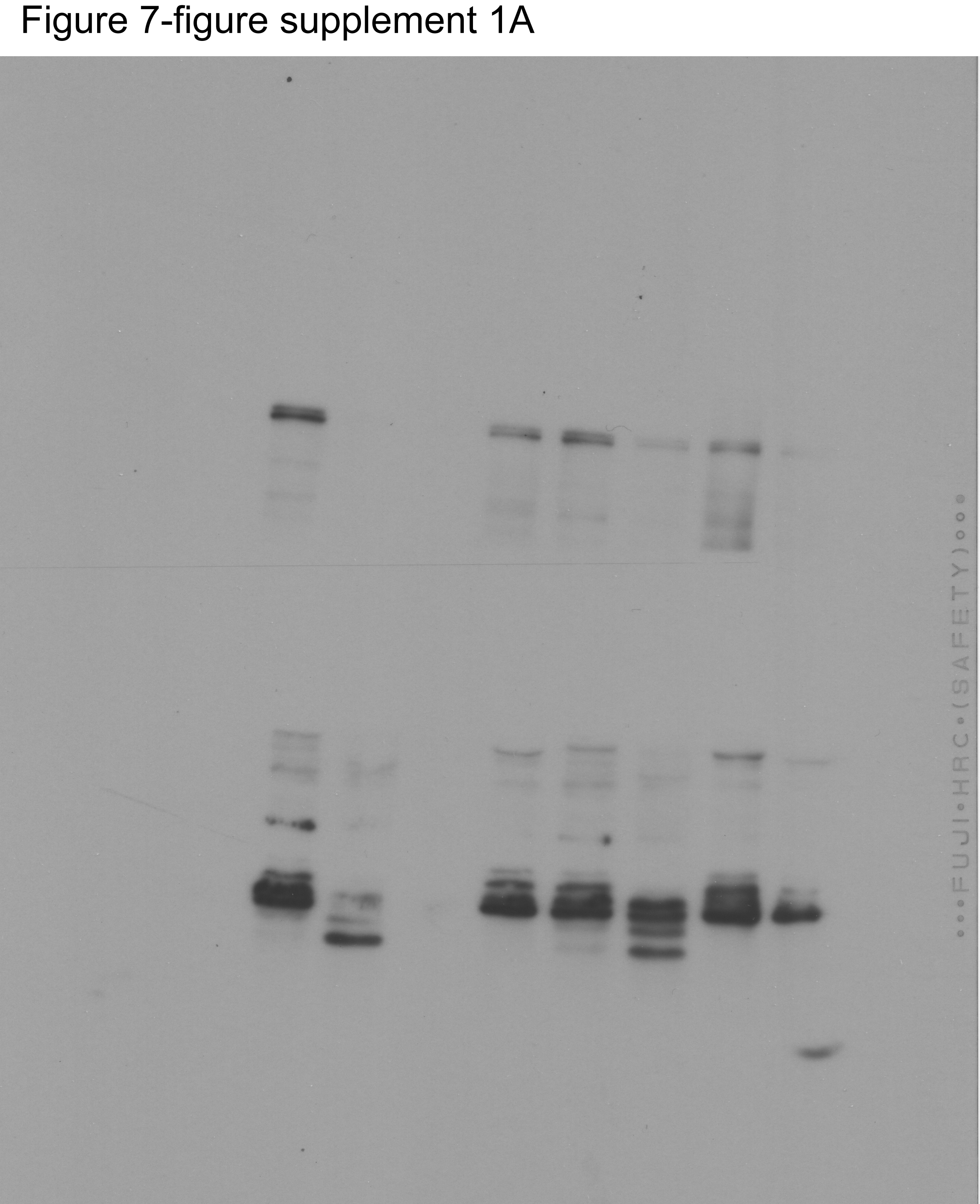

Supplement: Figure 7—figure supplement 1—source data 2. — Unlabelled. [file elife-74940-fig7-figsupp1-data2.zip › Figure 7-figure supplement 1 - source data.tif]
